# A facile route to synthesize CdSe/ZnS thick-shell quantum dots with precisely controlled green emission properties: towards QDs based LED applications

**DOI:** 10.1038/s41598-019-48469-7

**Published:** 2019-08-19

**Authors:** Junjie Hao, Haochen Liu, Jun Miao, Rui Lu, Ziming Zhou, Bingxin Zhao, Bin Xie, Jiaji Cheng, Kai Wang, Marie-Helene Delville

**Affiliations:** 1 CNRS, University Bordeaux, ICMCB, UMR 5026, F-33608 Pessac, France; 2 Department of Electrical and Electronic Engineering, Southern University of Science and Technology, Shenzhen, 518055 China; 3 Institute of Applied Physics and Materials Engineering, University of Macau, Avenida da Universidade, Taipa, Macau China; 4 School of Materials Science and Engineering, Hubei University, Wuhan, 430062 China

**Keywords:** Electronic devices, Energy

## Abstract

In recent, the quantum yield (QY) and stability of green quantum dots (QDs) have been significantly improved. However, most of the progresses were achieved by using alloyed QDs, and the control of green emission QDs still remains challenging. Herein, we report a novel method for synthesizing thick-shell structure quantum dots (TSQDs) with saturated green-emitting where tri-n-octylphosphine (TOP) was used as both ligand *and* solvent to extract the redundant ions from the QDs surface and remove the lattice imperfections before any surface inorganic layer-by-layer coating. The as-prepared TSQDs demonstrate enhanced luminescent properties including high QY reaching up to 75%, full width at half maximum (FWHM) remaining close to 26 nm and tunable precise emission properties (532 nm), which can be utilized to perform 91% of the International Telecommunication Union (ITU) Recommendation BT. 2020 (Rec. 2020) for high definition and color gamut displays.

## Introduction

Semiconductor quantum dots (QDs), thanks to their tunable emission property, have been broadly applied in light emitting diodes (LEDs) displays^1–5^, solar cells^6–8^ and biomedical applications^9–11^, etc. In these applications, it is a prerequisite to improve their chemical stability and photoluminescence (PL) quantum yield (QY) without changing their characters of facile tunable emission and narrow full width at half maximum (FWHM). To realize these targets, thick-shell QDs (TSQDs) were usually synthesized by epitaxial growth of inorganic shells on the cores with a consequent removal of the surface defects states^12^. In this way therefore, QDs with pure color emission of FWHM less than 30 nm can be obtained, accordingly achieving QD-based display devices with a promising color gamut^13^.

According to previous literature data, CdSe QDs with thick CdS shells exhibit reliable photoluminescence (PL) properties for saturated red emitting from 630 nm to 650 nm, with QY up to 97%, and FWHM of approximately 30 nm^1,6,14–16^. However, it still remains challenging to obtain efficient saturated green-emitting ranging from 520 nm to 540 nm for CdSe based TSQDs due to the following two main factors: (i) the large surface-to-volume ratio (S/V) which increases as the size decreases proportionally to 3/r, so that QDs are more sensitive to their environment and their PL intensity is easier to quench owing to more surface trap states^17^; (ii) it is not only difficult to fabricate such small CdSe semiconductor NCs with high QY and uniform size distribution, but also to achieve high stability by coating an inorganic semiconductor shell with wider-band-gap^18,19^. The conventional routes for synthesis of green-emitting QDs were basically relying on expensive reagents, such as 1-tetradecylphosphonic acid (TDPA), which form strong binding constant with cadmium ions resulting in a slow formation of QDs, and a subsequent small size of the resulting nanoobjects with a homogeneous size dispersions^3,20^. Up to now, none of these QDs were used to form thick-shell green-emitting QDs. Other attempts were based on alloyed QDs, such as ZnCdSe^21,22^, ZnCdSSe^18,23,24^, which provided large-sized QDs with green emission, however a systematic control over the emission wavelength of these QDs was extremely difficult not to say impossible. Thus, it is still mandatory and relevant to find out an effective synthetic approach to achieve TSQDs with pure green emission, high PL, QY, narrow FWHM and tunable precise emission properties.

We previously showed that tri-n-octylphosphine assisted successive ionic layer adsorption and reaction (TOP-SILAR) method was an efficient route to form shells with a precise thickness on QDs core and that thick-shell QDs had been successfully synthesized and were able to provide an accurate controllable emission in a wide color range, with a high QY and a narrow FWHM^25,26^. Core/shell QDs in the 570–615 nm range were synthetized this way.

**Figure 1:**
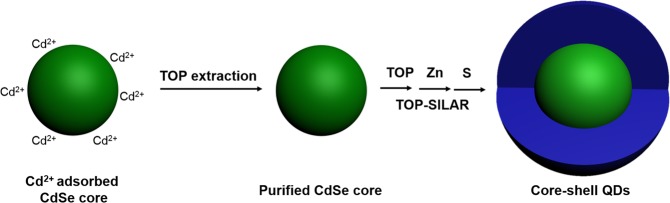
The growth scheme of modified TOP-SILAR method coupled with TOP extraction process using Cd^2+^ adsorbed CdSe core.

Herein, we improve this TOP-SILAR method with a TOP-assisted extraction process (Fig. 1) to fabricate CdSe/ZnS TSQDs which would extend this wavelength range down to the green emission (532 nm) with the same accuracy. This TOP-assisted extraction method used the excellent dissolving capability^14,27^ of the TOP ligand to clean-up the QD core surface before proceeding to the layer-by-layer coating. The resulting as-prepared TSQDs demonstrated a high quantum yield (QY) close to 75% and a narrow FWHM of 26 nm, corresponding to Commission Internationale de L’Eclairage (CIE) color coordinates of (0.194, 0.763), indicating a highly color-saturated emission, which is well suitable for an ultrahigh-definition (UHD) display application based on Rec. 2020 standard.

## Results and Discussion

The successive ionic layer adsorption and reaction (SILAR) is the most widely used technique to synthesize core/shell QDs^28^. We introduced tri-n-octylphosphine (TOP) in this shell growth process, which is named as TOP-SILAR method. TOP served as both a surfactant and solvent for synthesizing nanoparticles, playing a crucial role in not only dissolving and activating the precursors but also controlling the size, morphology and stability of the nanoparticles, breaking through the bottleneck of low QY at high coverage of the shell^26^. The TOP-assisted extraction method is based on the TOP-SILAR method with some modification. The QDs core was first purified by the TOP-assisted extraction process, which adding TOP to the hexane/methanol extraction system, and thus more effective purify the incomplete reaction of the precursors. And then the TOP-SILAR process was introduced for further growth of core/shell QDs.

In this work, we have successfully synthesized green-emitting thick-shell CdSe/ZnS QDs via a modification of the TOP-SILAR method^26^. The synthesis process is presented graphically in Fig. 1, the small size CdSe cores were prepared by a swift cooling process in order to generate cores as small as possible. The slow reaction rate of CdSe generation and the swift quenching of the temperature provide tiny dots, however the reaction is far from complete. This is why this first step had to be followed by a so-called TOP-assisted extraction process, it is assumed that this process can efficiently eliminate the excess of Cd^2+^ and the non-nanocrystalline side-products present on the CdSe core or in solution, and which might affect the subsequent TOP-SILAR technique which allowed the *in-situ* ZnS growth using TOP as an activator and led to the CdSe/ZnS core/shell TSQDs. Since TOP molecules were present all along the shell growth process, they could not only efficiently dissolve the isolated ZnS nanocrystals which might have formed and but also remove any surface defects of the core/shell QDs, so as to achieve nanodots with a narrow emission spectral width.

**Figure 2:**
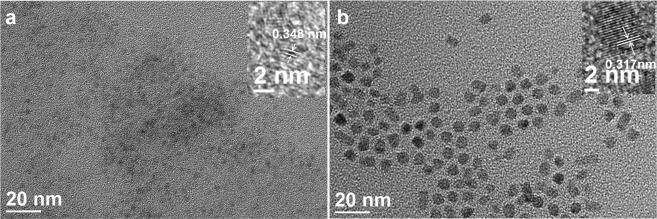
TEM and HRTEM (inset) images of (**a**) bare green-emitting CdSe cores (2.5 nm); (**b**) corresponding green-emitting CdSe/ZnS(7) thick-shell QDs (7.2 ± 0.9 nm), lattice fringes with interlayer distance of 0.348 nm, and 0.317 nm are displayed. The crystallinity is confirmed by the distinct lattice fringes.

Figure 2(a,b) show the TEM images and high resolution images (insets) of the as-prepared green-emitting CdSe cores and CdSe/ZnS(7) TSQDs respectively. TOP-assisted extraction process successfully preserved the size distribution and mono-dispersity of the QDs cores and the shell formation process. Furthermore, the ZnS shell thickness can be estimated by the subtraction of the core size from that of the present prepared core/shell particles. The diameter of the CdSe particles (2.5 nm) was calculated using the empirical formula developed by Peng *et al*. (SI Equation 1)^20^. The layer-by-layer nature of the TOP SILAR technique provides a nice control of the ZnS thickness around the CdSe core. As an example, the average diameter of CdSe/ZnS(7) TSQDs, as measured by transmission electron microscopy (TEM), is 7.2 nm (±0.9 nm) (Fig. S1(d2)), leading to an average shell thickness of 4.7 nm (±0.9 nm), which consequently corresponds to 7 monolayers (MLs) of shells (1 ML of ZnS was approximated to be a 0.7 nm increase in diameter/size as estimated previously)^2,28,29^. Core/shell QDs with 1, 3 and 5 layers are also shown in Fig. S1. The insets in Fig. 2(a,b) show high-resolution TEM (HRTEM) images of representative single nanocrystals. It is revealed that the whole particles show high crystallinity with continuous lattice fringes, and between the core and shell no evidence of interface can be seen. This means that the shell growth process occurs in the case of coherent epitaxial region, and excludes the independent homogeneous nucleation of shell precursors^30^. We can see from the inset in Fig. 2a, 0.348 nm of interplanar spacing was derived for the CdSe core QDs, which is in good agreement with the crystal lattice plane (111) of bulk cubic zinc blende (ZB) CdSe (d_111_ = 0.351 nm for ZB CdSe, JCPDS No. 00-019-0191)^31–34^. As shown in the HRTEM image of CdSe/ZnS(7) TSQDs (inset in Fig. 2b), the distance of interplanar spacing is 0.317 nm, which is in good agreement with cubic ZnS in the lattice spacing of the (111) planes (0.312 nm)^31,35^.

**Figure 3:**
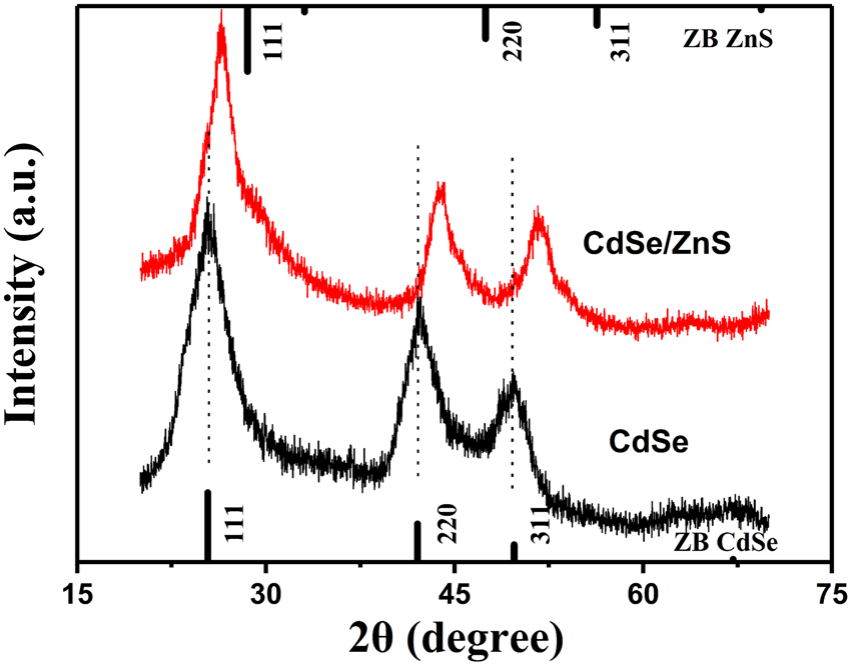
Powder X-ray diffraction (XRD) patterns of green-emitting CdSe and CdSe/ZnS(7) TSQDs as shown in Fig. 2. The XRD peaks position of the zinc blende (ZB) bulk CdSe (JCPDS No. No. 00-019-0191) and bulk ZnS (JCPDS No. 80-0020) are respectively indicated by vertical solid lines below and above. Dash lines are a guide to the eye.

In parallel, X-ray diffraction (XRD) measurements were employed to examine the crystal structure of the TSQDs. The XRD pattern of CdSe shows peaks at 25.3, 42.1, and 49.7 degrees, which correspond to the lattice planes of (111), (220), and (311) (Fig. 3), respectively. These results are completely in conformity with the standard JCPDS No. 00-019-0191^18^. There are peak shifts in large angle direction and peak shrinks with the ZnS shells growing on the zinc blende CdSe cores. It is also clearly shown that as the thickness of the shell increases, the XRD peaks become narrower, indicating that the QDs are getting bigger, which demonstrates the successful epitaxial growth of the ZnS shell^26,36,37^.

**Figure 4:**
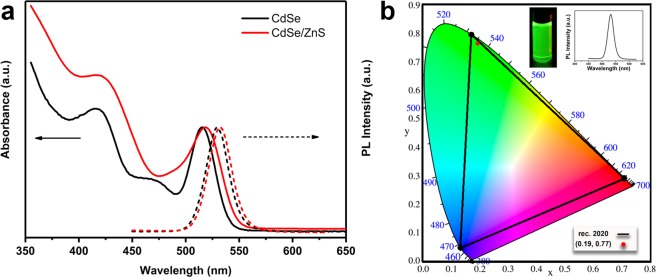
(**a**) PL spectra and absorption spectra of green-emitting CdSe cores and green-emitting CdSe/ZnS(7) TSQDs; (**b**) The corresponding CIE coordinates of green-emitting CdSe/ZnS(7) core/shell QDs synthesized by modified TOP-SILAR method using TOP extraction process. (Insets: as prepared QDs and the PL spectrum).

The normalized PL spectra of QDs are shown in Fig. 4(a). The first exciton absorption peak and the emission peak of the synthesized CdSe cores are 516 nm and 529 nm, respectively. After the growth of ZnS thick-shells, the first exciton absorption peak and the emission peak of the TSQDs slightly red-shifted to 519 nm and 532 nm, respectively. In addition, the FWHM of PL peaks maintained the same (25 nm and 26 nm), and the QY value consequently increases from an initial 21% to a final 75%. The PL emission spectra of CdSe/ZnS core/shell QDs exhibit a slight red shift with the shell growth as compared with that of the CdSe QD core, and the FWHM of the obtained core/shell QDs remains very narrow (Fig. S2). Based on the emitting wavelength and FWHM obtained from their PL spectrum, these TSQDs exhibit the following color coordinates (0.194, 0.763) (Fig. 4(b)), which almost correspond to the ideal saturated green point (0.170, 0.797) in ITU-R Recommendation BT.2020 (Rec. 2020) posted in 2012 for the new generation of high definition displays^38–41^.

**Figure 5:**
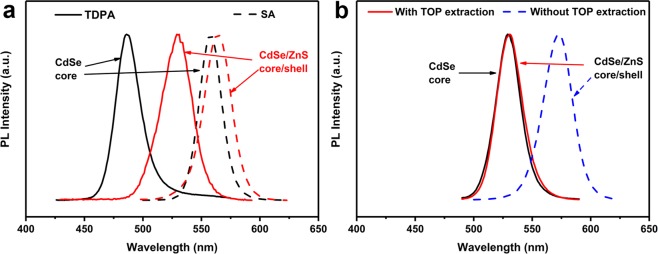
(**a**) Normalized PL spectra of CdSe/ZnS(3) core/shell QDs synthesized by different precursors, using Cd(TDPA)_2_ (solid line, inadequate reaction) and cadmium stearate (Cd(SA)_2_) (dashed line, adequate reaction) as CdSe precursors; (**b**) Normalized PL spectra of QDs in different shell formation synthetic processes, CdSe/ZnS(3) core/shell QDs synthesized by typical TOP-SILAR method based on the rapid cooling process core (black solid line), with TOP-assisted extraction purification process (red solid line), without TOP assisted extraction purification process (blue dashed line).

To probe the mechanism for the synthesis of green-emitting TSQDs, the precursor effect was firstly investigated: two commercially available precursors (TDPA expensive and stearic acid (SA) cheap) were used to generate respectively Cd(TDPA)_2_, and Cd(SA)_2_ as precursors of the green-emitting QD cores. The later were then used in a typical TOP-SILAR method synthesis of CdSe/ZnS core/shell QDs. When TDPA was used as a ligand to synthesize the core *without* the TOP assisted extraction purification process (Fig. 5(a), solid line), a large amount of metal ion precursors and non-nanocrystalline side products still co-exist in the reaction system due to the low reaction rate^42,43^, resulting in a uncontrollable red-shift of more than 40 nm after the typical TOP-SILAR shell growth process. While in the case of a SA ligand, the fabrication of CdSe core seems to be easier and faster and we assumed that there’s no excess of Cd^2+^ remained (Fig. 5(a), dashed line)^44^, showing only a small red-shift of 6 nm after a typical shell growth, however it is still difficult to prepare a fully reacted CdSe QDs with a wavelength less than 550 nm.

From now on we worked with Cd(SA)_2_ as unique precursor and showed the positive influence of the TOP *extraction* method as illustrated in Fig. 5(b). In this case, the green-emitting CdSe cores were generated using a cheap reagent (SA) and a rapid cooling process. They were then submitted (red line) or not (dashed blue line) to a TOP-assisted extraction prior to the TOP-SILAR method process. The resulting CdSe/ZnS core/shell exhibits a green emission which successfully overlays that of the core when TOP extraction is used, we assumed that (i) this step is crucial to efficiently remove the excess amount of Cd^2+^ ions and non-nanocrystalline side products whether they are on the surface of the CdSe core (as shown in Fig. 1) or in solution, and (ii) the CdSe/ZnS core/shell QDs can effectively be obtained by this layer-by-layer technique without modification of the emission maximum^25^. As a comparison (Fig. 5b dashed line and Fig. S3), when no TOP extraction process is applied before the typical TOP-SILAR method, a significant red shift of the emission peak takes place along this process, it is assumed that this is due to the large excess of Cd^2+^ and other non-nanocrystalline side products in the medium which remain trapped in the shell during its formation. Since it is very hard to quantitatively analyze the excess amount of Cd^2+^ ions and of species of the side products, it is also hard to evaluate the thickness of the shell and the emission properties of the core/shell QDs often encounters problems with reproducibility, justifying once more a real need for a purification process leading to reliable QDs. The detailed emission peak evolution during the TOP extraction process can be seen in Fig. S4, the red-shift gradually decreases as the TOP assisted extraction purify process proceeds. As using the same batch of CdSe cores, the shell growth process based on the TOP extraction method was repeated several times, and the results are shown in Table S1. The emission peak, the FWHM and the QY show a high level of reproducibility, which is controlled at a variation range ±1.5 nm, ±1.2 nm, and ±2%.

These results reveal that the traditional purification processes (hexane/methanol, toluene/methanol) did not provide a high enough level of performance in terms of cleaning ability and they were unable to prevent secondary reactions of the core precursors^42^. Moreover, there is a real need of the effective elimination of redundant Cd^2+^ since it reduces the lattice mismatch between the core and shell materials, which is greatly beneficial to improve the QY of the final TSQDs. The extraction process is, thus, proved to be a key factor in controlling the emitting wavelength of the TSQDs especially in the green region (<550 nm) (corresponding to the smallest CdSe core). This TOP extraction process is of course general and can also provide red core/shell QDs with good emission, as shown in Fig. S5, in which the emission maximum moved from 632 nm to 634 nm during the whole process, and the FWHM of PL peaks can be maintained (26 nm and 28 nm). The TEM images and high resolution images (insets) of the red-emitting CdSe cores and CdSe/ZnS core/shell QDs are shown in Fig. S6 respectively. The average diameter gradually increases from 5.6 nm (±0.5 nm) to 7.7 nm (±1.0 nm) (Fig. S7). Additionally, if these green-emitting TSQDs are combined with red ones, they form hybrid spectrum which can be used to achieve around 91% Rec. 2020 high color gamut display devices (Fig. S8), this is of great implications for the development of advanced display devices.

**Figure 6:**
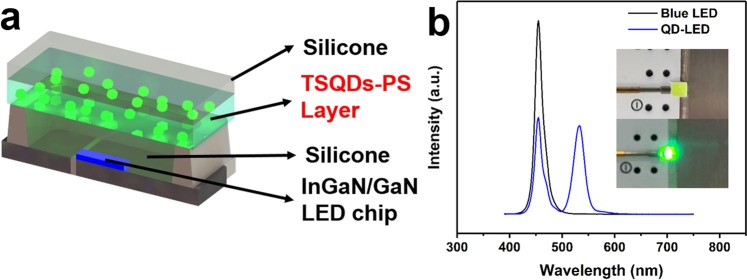
(**a**) Schematic of a remote packaging LED with green-emitting TSQDs; (**b**) the EL spectra of a blue LED and a green QD-LED operated at a current of 20 mA at 2.7 V, the inset is the photograph of green-emitting LED under daylight and operation at 20 mA.

The photoluminescence (PL) of QDs is known to gradually drecrease as the temperature incresases and is difficult to recover after high temperature heating process^45,46^. In order to simulate the effect of temperature on quantum dots, we placed the green-emitting QDs at different temperatures to study the stability of quantum yield (Fig. S9). The PL QY decreased to 35% after 4 h heating at 200 °C. Considering the blue LED chips can generate much heat, which can damage to QDs, an effective method is adopted to control this phenomenon^47–49^. This approach separated QDs layer from the blue LED excitation light source, so that the temperature in QDs layer is much lower due to the cooling effect of the space between LED excitation light source and QD layer^47,50^, as shown in Fig. 6(a), the detailed fabrication method for the green-QD-LEDs is described in the Supporting Information. Firstly, an independent green-emitting-QDs-PS composite plate was prepared using a green-emitting CdSe/ZnS TSQDs with QY of 75%. The polystyrene (PS) polymer particles dissolved in chloroform were mixed with green-emitting QDs, and then the QD-PS composite plate was obtained after natural curing under an inert atmosphere. A photograph of the flexible QD-PS composite plate with high fluorescence under a 365 nm UV irradiation is shown in Fig. S9. Then, a QD-based remote-type LED was simply prepared by cutting the plate into a suitable size and combining it with silicone resin (silicone 6550 gel A/gel B = 1:1) on an In-GaN/GaN blue LED chip (455 nm). As shown in Fig. 6(b), both the blue LED and the QD-LED spectra were operated at a current of 20 mA at 2.7 V. The emission of the TSQDs layer was slightly shifted to 533 nm and the FWHM was as narrow as 25 nm, indicating the achievement of a green LED. The photo of a green-LED operated at a driving current of 20 mA is shown in Fig. 6(b) (insert). This also demonstrates that our TSQDs can be easily applied in QD-LEDs.

## Conclusion

In conclusion, we reported an improved facile synthesis method to synthesize CdSe/ZnS thick-shell QDs with reproducible saturated green and high luminance emission. The cores are generated through a rapid cooling process using cost-effective reagents (SA and CdO). The shell is build-up on a Lego-type basis associating a TOP extraction method to clean-up the QD surface, and a TOP-SILAR method. QDs with a high QY of 75%, a narrow FWHM of 26 nm and a controllable emission can be obtained and the green emission (532 nm) can be maintained even after coated with a thick ZnS shell (7 monolayers). This inexpensive and high-output method can also be utilized to obtain multi-color core/shell QDs with high precision. In addition, the experimental results obtained in this research are beneficial to understand the mechanism of TOP-assisted method, and provides (i) real new access to QDs with properties exhibiting a high level of reproducibility in terms of size, purity, wavelength and QY (ii) a promising low-cost way to mass fabrication of LED as the pure-colored backlight in display.

## Methods

### Chemicals

Cadmium oxide (99.99%), zinc oxide (99.99%, powder), stearic acid (SA, 95%), selenium powder (99.5%, powder), oleic acid (OA, 90%), and sulfur (99.5%, powder)were obtained from Sigma, USA. 1-octadecene (ODE, 90%), tri-n-octylphosphine (TOP, 97%), octadecylamine (ODA, 90%), trioctylphosphine oxide (TOPO, 90%), and other organic solvents were purchased from J & K Chemical Reagent Company, China. All the chemicals were used as received without further purification.

### Synthesis of green-emitting CdSe core by stearic acid through rapid cooling process

The synthetic procedure was based on the procedure from the literature except for the rapid cooling process after the Se-TOP injection^26,44,51^. Typically, 0.4 mmol of stearic acid and 0.2 mmol of CdO were mixed in a 25-mL three-neck flask, which was heated to 220 °C so as to obtain a colorless clear solution under argon atmosphere. And then the mixture was allowed to cool to room temperature, ODA (2 mmol, ODA:Cd = 10:1) and ODE (8 ml) were sequentially added into the flask, and the mixture was reheated to 270 °C under inert atmosphere. As soon as the heating device removed, 2 ml of Se-TOP (1 M) and 4 ml of ODE were injected sequentially (rapid cooling process 10 to 20 s). At last, the reaction mixture was cooled to less than 60 °C, and the nanocrystals were purified by a typical hexane/methanol extraction procedure^28,44,52^. The obtained green-emitting CdSe cores (529 nm, FWHM 25 nm) were dispersed in hexane.

### TOP assisted extraction process

4 ml of ODE was added to the CdSe-hexane solution, the mixture was pumped at room temperature to remove the hexane and then further pumped at 100 °C to remove any residual air from the system^25^. After the argon atmosphere was switched to the system, 0.5 ml of TOP were injected as an activator, and the mixture solution was further heated to160 °C for 10 min^51^. Then the solution was allowed to cool to less than 60 °C, and the typical hexane/methanol (volume ratio: 1:2) extraction procedure was used for the further purification. The TOP assisted extraction process was repeated 3 times, and then the purified nanocrystals dispersed in the hexane/ODE mixture phase were used for shell growth process, and the adsorbed Cd^2+^ maybe remain in the alcohol phase and could be removed.

### Synthesis of green-emitting thick-shell core/shell QDs by TOP-SILAR method

High quality green-emitting CdSe/ZnS core/shell QDs can be synthesized by modified tri-n-octylphosphine-assisted successive ionic layer adsorption and reaction (TOP-SILAR) described in our precious research papers^25,26,53^, using the TOP assisted extraction process purified CdSe as cores. The obtained green-emitting TSQDs (7-monolayered ZnS shell) with emission peak at 532 nm, and 26 nm for FWHM.

### Fabrication of QDs-PS composite plate

The physical mixing method was used to form a polymer plate with green-emitting TSQDs as in the refs^4,54^. Typically, polystyrene (PS) polymer particles (1 g) was dispersed in chloroform (3 ml), and then mixed with the prepared QDs solution (1 ml). The QDs-PS composite plate was prepared after drying in the mold for more than 20 h under argon atmosphere, and then compression molding by a vulcanizing machine (4 MPa) at 100 °C for about 10 min.

### Assembling of TSQDs-LED

The blue In-GaN/GaN LEDs with the peak emission at 455 nm were used. The QDs-PS composite plate was first mixed with silica gels (silicone 6550 gel A/gel B = 1:1) and then pasted onto the SMD-type LED. The LED chip was allowed to cure at 100 °C (30 min) so as to obtain the TSQDs based LED^4^. The EL spectra were tested in an integrated sphere and a spectrograph system.

Further details of the syntheses are provided in the Supporting Information, along with details of the characterization techniques.

## Supplementary information

Supplementary Material
