# Supplementary material for: A facile route to synthesize CdSe/ZnS thick-shell quantum dots with precisely controlled green emission properties: towards QDs based LED applications
Source: Sci Rep. 2019 Aug 19;9:12048. doi: 10.1038/s41598-019-48469-7 (PMC6700096; doi:10.1038/s41598-019-48469-7)
Supplement: Supplementary file 1 — Supplementary Material [file 41598_2019_48469_MOESM1_ESM.pdf]

Electronic Supplementary Information

A facile route to synthesize CdSe/ZnS thick-shell quantum dots with precisely controlled green emission properties: towards QDs based LED applications

Junjie Hao,[1,2] Haochen Liu,[2]Jun Miao,[3] Rui Lu,[2] Ziming Zhou,[2] Bingxin Zhao,[2] Bin Xie,[2] Jiaji Cheng,[4] Kai Wang,[2*] & Marie-Helene Delville[1*]*

[1]*CNRS, Univ. Bordeaux, ICMCB, UMR 5026, F-33608, Pessac, France*

[2]*Department of Electrical and Electronic Engineering, Southern University of Science and Technology, Shenzhen, 518055, China*

[3]*Institute of Applied Physics and Materials Engineering, University of Macau, Avenida da Universidade, Taipa, Macau, China*

[4]*School of Materials Science and Engineering, Hubei University, Wuhan 430062, China*

Corresponding author: sosjune@163.com; wangk@sustc.edu.cn; marie-helene.delville@icmcb.cnrs.fr

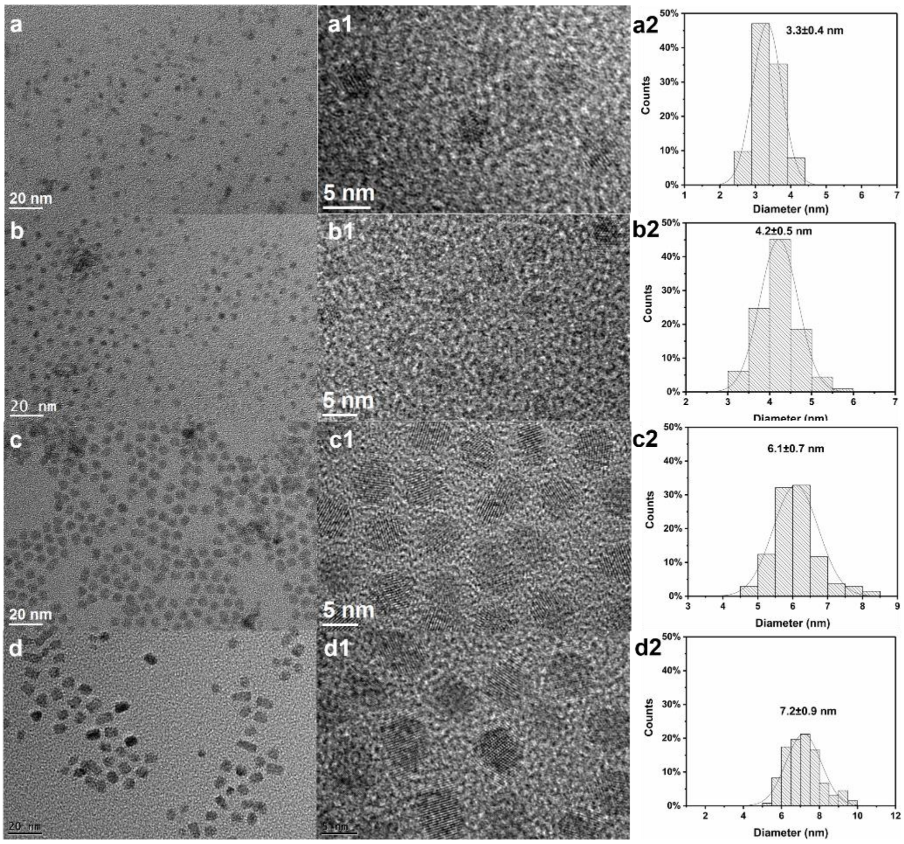

Figure S1. TEM of CdSe/ZnS TSQDs with the increase of shell thickness: (a) 1 layer of ZnS shell; (b) 3 layer of ZnS shell; (c) 5 layer of ZnS shell; (d) 7 layer of ZnS shell. (a1-d1): Corresponding HRTEM of parts a-d. (a2-d2): Corresponding histogram of the diameter distribution of parts a-d.

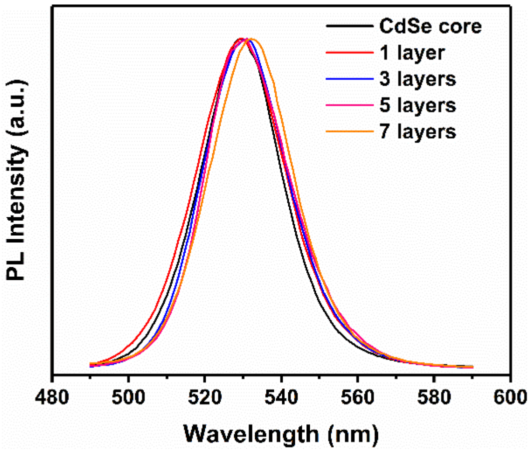

Figure S2. Normalized fluorescence spectra of CdSe/ZnS QDs with the increase of shell thickness: CdSe core (black), 1 layer of ZnS shell (red), 3 layer of ZnS shell (blue), 5 layer of ZnS shell (magenta), and 7 layer of ZnS shell (orange). Their corresponding full-width at half-maximum (FWHM) is 25 nm, 28 nm, 28 nm, 26 and 26 nm, respectively.

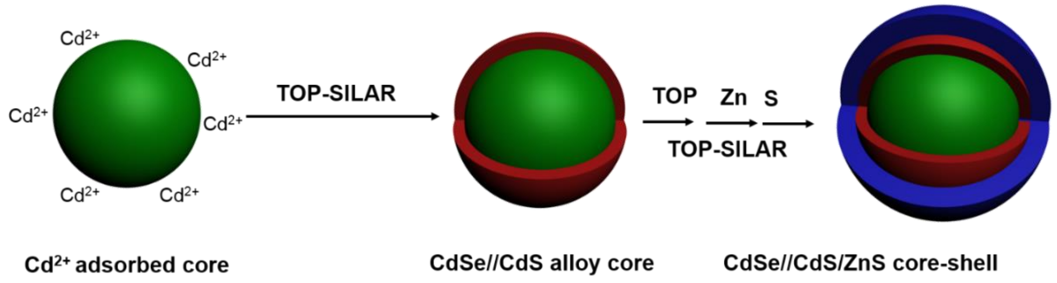

Figure S3. The growth schematic of typical TOP-SILAR method using Cd^{2+} adsorbed CdSe core.

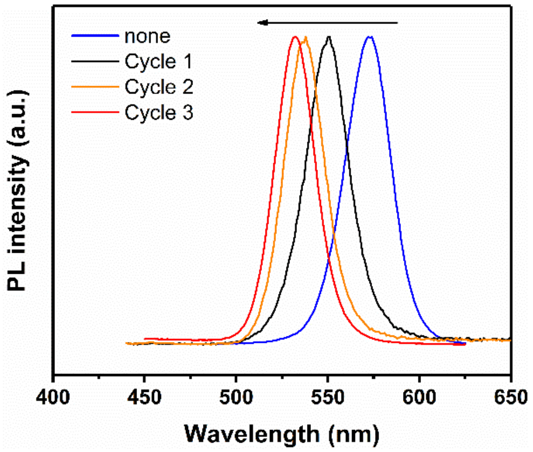

Figure S4. Fluorescence spectra of core-shell QDs synthesized by TOP-SILAR method. Using the rapid cooling CdSe as core, a 3 -monolayered ZnS shell was grown under different purification process. From left to right: TOP assisted extraction purify process 3 times; TOP assisted extraction purify process 2 time; TOP assisted extraction purify process 1 time; typical hexane/methanol extraction procedure.

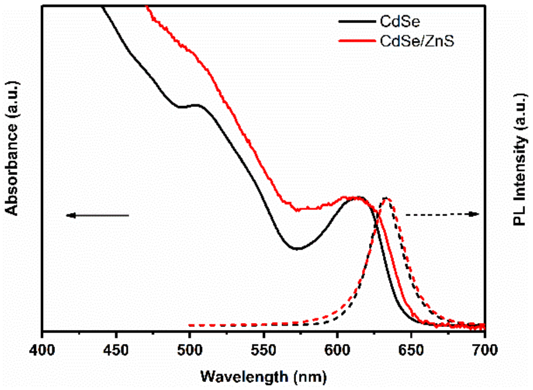

Figure S5. Fluorescence spectra and absorption spectra of the red CdSe core (FWHM 26 nm) and red CdSe/ZnS(3) core/shell QDs (FWHM 28 nm) synthesized by TOP-SILAR method, coupling with TOP assisted extraction purification process.

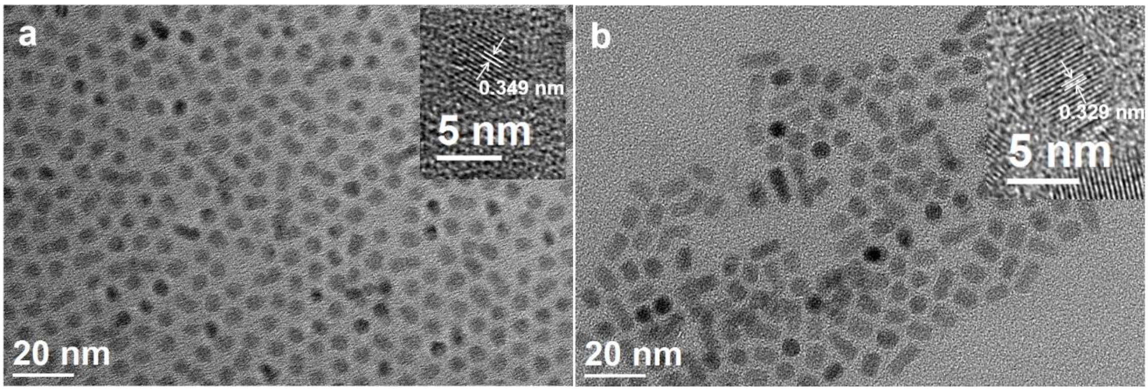

Figure S6. High resolution TEM images of (a) bare red CdSe cores; (b) corresponding red CdSe/ZnS(3) core-shell QDs, lattice fringes with interlayer distances of 0.349 nm, and 0.329 nm are displayed.[1] The diameter of the bare core CdSe nanocrystals is 5.6 nm, and 7.7 nm for the core-shell QDs. The stripes are all in the same direction, indicating that the nanocrystal is single crystalline. The scale bar is 20 nm, and the insert scale bar is 5 nm.

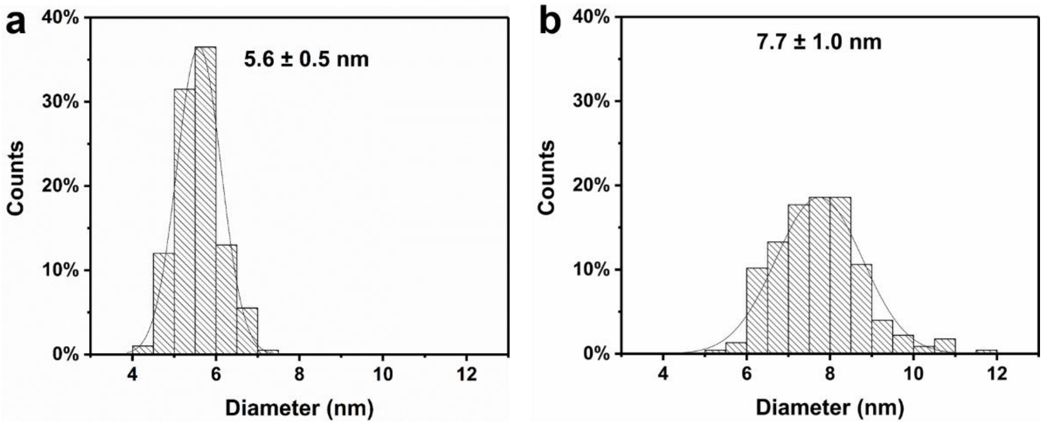

Figure S7. Histograms of the diameter distribution of red QDs (a) and red core-shell QDs (b) as shown in Figure S5.

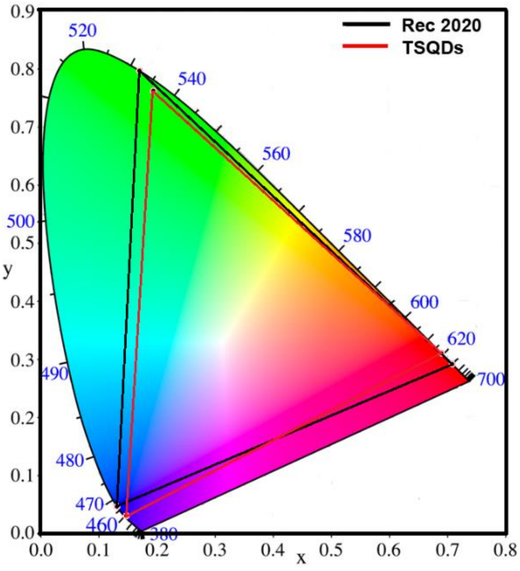

Figure S8. The corresponding CIE coordinates of green-emitting and red-emitting QDs synthesized by modified TOP-SILAR method using TOP extraction process. The TSQDs can be utilized to realize about 91% Rec. 2020 high color gamut display devices.

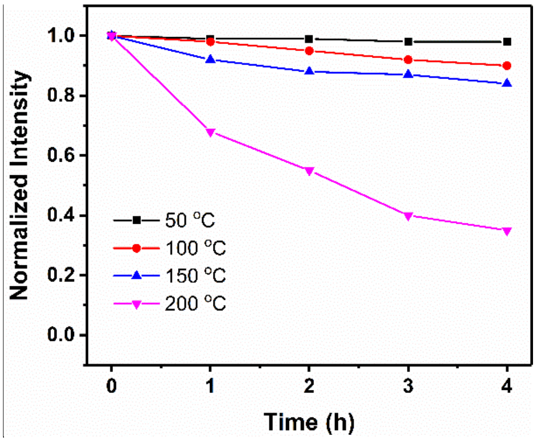

Figure S9. Temperature-dependent photoluminescence quantum yield (PL QY) of green-emitting QDs.

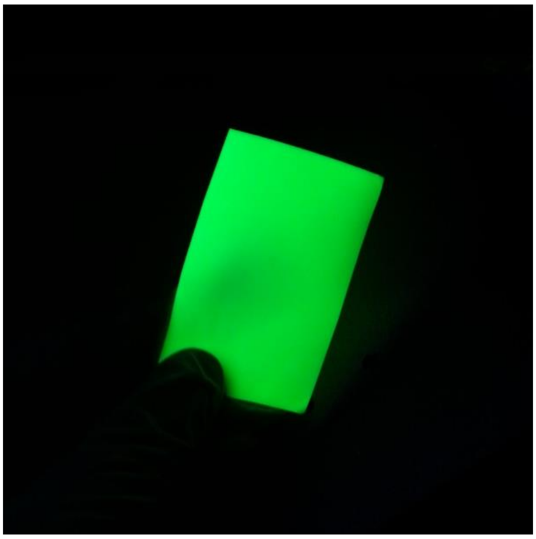

Figure S10. The photograph of a free-standing green-emitting QDs-PS composite plate under a 365 nm UV illumination.

Table S1. Reproducibility of shell growth process for the TOP-assisted extraction method

Sample No.	Core wavelength (nm)	Core-shell wavelength (nm)	FWHM (nm)	QY (%)
1	529	532	26	75
2	529	533	26	70
3	529	535	28	73
4	529	531	25	72
5	529	533	25	70
Average	-	533 ± 1.5	26 ± 1.2	72 ± 2

1. Experimental Procedures

1.1 Chemicals

Cadmium oxide (CdO) (99.99%), zinc oxide (99.99%, powder), stearic acid (SA, 95%), selenium powder (Se) (99.5% powder), oleic acid (OA, 90%), and sulfur (S) (99.5%, powder) were obtained from Sigma, USA. 1-octadecene (ODE, 90%), tri-n-octylphosphine (TOP, 97%), octadecylamine (ODA, 90 %), and trioctylphosphine oxide (TOPO, 90%), and other organic solvents were purchased from J & K Chemical Reagent Company, China. All the chemicals were used as received without further purification.

1.2 Synthesis of green-emitting thick-shell core-shell QDs

1.2.1 Synthesis of green-emitting CdSe core by stearic acid through rapid cooling process

The synthetic procedure was based on the procedure in the literature except for the rapid cooling process after the Se-TOP injection.[2-4] Typically, 0.4 mmol of stearic acid and 0.2 mmol of CdO were mixed in a 25-mL three-neck flask, which was heated to 220 °C so as to obtain a colorless clear solution under argon atmosphere. And then the mixture was allowed to cool to room temperature, ODA (2 mmol, ODA:Cd=10:1) and ODE (8 ml) were added into the flask, and the mixture was reheated to 270 °C under argon atmosphere. As soon as the heating device removed, 2 ml of Se-TOP (1M) and 4 ml of ODE was injected sequentially over a period of 5-10 s (rapid cooling process). Finally, the reaction mixture was cooled to less than 60 °C, and a typical hexane/methanol extraction procedure was used to purify the nanocrystals.[2,5] The obtained green-emitting CdSe core (529 nm, FWHM 25 nm) was dispersed in hexane.

1.2.2 TOP assisted extraction process

4 ml of ODE was added to the CdSe-hexane solution, the mixture was pumped at room temperature to remove the hexane and then further pumped at 100 °C to remove any residual air from the system.[6] After the argon atmosphere was switched to the system, 0.5 ml of TOP was injected as an activator, and the mixture solution was further increased to 160 °C for 10 min.[4] Then the solution was allowed to cool to less than 60 °C, and the typical hexane/methanol (volume ratio: 1:2) extraction procedure was used for the further purification. The TOP assisted extraction process was repeated 3 times, and then the purified nanocrystals dispersed in the hexane/ODE mixture phase were used for shell growth process, and the adsorbed Cd^{2+} maybe remained in the alcohol phase and could be removed.

1.2.3 Calculations for the Injections

The amount of CdSe seeds was determined with absorption spectroscopy using the empirical formulas of Peng *et al.*[7] which correlates the position of the first electronic transition with the diameter of the CdSe particles (equation 1) and the molar extinction coefficient of the particles and their diameter (equation 2).

$$D = (1.6122\times10^{-9})\cdot\lambda^4 - (2.6575\times10^{-6})\cdot\lambda^3 + (1.6242\times10^{-3})\cdot\lambda^2 - (0.4277)\cdot\lambda + (41.57) \quad (1)$$

$$\varepsilon=5857\cdot(D)^{2.65} \quad (2)$$

The SILAR technique is based on the alternating injections of the Zn and S precursors into the solution containing CdSe nanocrystals for the growth of CdSe/ZnS core/shell nanocrystals. The amount of zinc or sulfur precursors required for each layer was determined by the number of the surface atoms of a given size of a core/shell nanocrystal. We estimated the number of monolayers assumimg each layer of ZnS had a thickness of 0.35 nm, so one additional layer growth would increase the diameter of a nanocrystal by 0.7 nm.[8]

For example, in a typical experiment with 1×10^{-5} mmol of 2.5 nm core, 2.27×10^{-3} mmol of Zn and S precursors is needed

for the first layer of the shell growth, and an additional 3.51 × 10^{-3} mmol of Zn and S precursors completes the growth of the second layer.

1.2.4 Synthesis of green-emitting thick-shell core-shell QDs by TOP-SILAR method

High quality green-emitting CdSe/ZnS core-shell QDs can be synthesized by modified tri-n-octylphosphine-assisted successive ionic layer adsorption and reaction (TOP-SILAR) described in our precious research,[3,6,9] using the TOP assisted extraction process purified CdSe as core.

For a typical reaction, the QDs dissolved in 2.5 mL of hexanes were mixed with 0.8 g of ODA and4.0 mL of ODE in a 25-mL three-neck flask. The flask was pumped down at room temperature with a mechanical pump for 30 min to remove the hexanes and at 100 ^oC for another 10 min to remove any residual air from the system. Subsequently, the system was switched to argon atmosphere and heated to 140 ^oC for the injections.

For in-situ growth of the buffer layer, 0.4 mL of TOP solution was injected as an activator and the reaction mixture was further maintained at 200 ^oC for 30 min. Then, calculated amount of Zn solution (0.1 M) was injected and maintained at 200 ^oC for 20 min.

After that, without further purification, the reaction temperature was decreased from 200 ^oC to 180 ^oC. Then the same amount of S precursor (0.1 M) solution was added into the reaction flask via syringe and the temperature was increased to 220 ^oC immediately, for the growth of ZnS outer-layers. After 60 min, the first ZnS monolayer was grown in-situ, and then the temperature was decreased to140 ^oC.

After 0.4 mL of fresh TOP solution was injected, the temperature was increased immediately to 180 ^oC. The Zn and Se precursor solutions (calculated for the second layer) were added consecutively via syringe to the reaction flask at the interval of 10 min for the growth of second ZnS monolayer. Cycling of injection and growth continued for both the increased monolayers of ZnS shell, the amounts of subsequent injection solutions were calculated using the method described in our previous work.[3] The reaction was terminated by allowing the reaction mixture to cool. The final product was diluted by hexanes followed by a methanol extraction. The extraction procedure was repeated for three times, and the top hexane layer was stored. If necessary, heating or further addition of ODE can be used for the better extraction. The nanocrystals were further purified by precipitating with acetone or methanol. Highly pure nanocrystals were obtained by repeating the above purification procedure for several times. The obtained green-emitting TSQDs (7-monolayered ZnS shell) with emission peak at 532 nm, and 26 nm for FWHM.

1.2.5 Synthesis of red core-shell QDs by TOP-SILAR method

The red CdSe/ZnS core-shell QDs was made by the same process as the green-emitting TSQDs using the 632 nm CdSe core (FWHM 26 nm).

As for the red emission CdSe QDs, 0.8 mmol of stearic acid and 20 mmol of ODA was used, and the reaction was maintained at 250 °C for 5 min. The CdSe was purified by TOP assisted extraction process (1.2.2), and then growth the shell as 1.2.4. The obtained red core-shell QDs with emission peak at 634 nm (FWHM 28 nm).

1.3 Synthesis of the CdSe//CdS/ZnS core-shell QDs

The CdSe//CdS/ZnS core-shell QDs were synthesized based on typical TOP-SILAR method using the green-emitting CdSe core (1.2.1) directly without TOP assisted extraction process.[6] The same process as 1.2.4 was used and the emission peak with 573 nm was obtained.

1.4 Synthesis of green-emitting CdSe/ZnS core-shell QDs using Cd(TDPA)$_2$ as core precursors

1.4.1 Synthesis of CdSe based on $Cd(TDPA)_2$

The comparison green-emitting CdSe/ZnS using $Cd(TDPA)_2$ as core precursors was synthesized as in the literature.[7,10,11] Typically, CdO and TDPA were mixed in ODE. The mixture was degassed, heated to 280 °C under argon atmosphere to obtain a colorless clear solution. The CdSe core QDs were prepared by quick injection of 1M of Se-TOP precursor into the Cd-containing reaction mixture at 300 °C. After 1 min, the reaction mixture was cooled to 50-60 °C. The obtained QDs with an emission peak at 486 nm, and FWHM 25 nm, and a typical hexane/methanol extraction procedure was used to purify.

1.4.2 *Synthesis of green-emitting CdSe/ZnS core-shell QDs*

The green-emitting core/shell QDs was synthesized based on typical TOP-SILAR method using the obtained CdSe core.[6] The same process as 1.2.4 was used and the emission peak with 530 nm was obtained.

1.5 Synthesis of CdSe/ZnS core-shell QDs by typical TOP-SILAR method using the CdSe without rapid cooling process

1.5.1 Synthesis of CdSe core by stearic acid without rapid cooling process

The highly fluorescent CdSe nanocrystals were prepared by the procedure in literatures as the QDs core.[2] The synthesis process was the same as Cd^{2+} adsorbed CdSe core (1.2.1) except for without rapid cooling procedure. Typically, a mixture of 0.2 mmol of CdO and 0.4 mmol of stearic acid in a 25-mL three-neck flask was heated to about 220 °C under argon atmosphere to obtain a colorless clear solution. After cooling to room temperature, 2 mmol of ODA (ODA:Cd=10:1) and 8 ml of ODE were added into the flask, and reheated to 270 °C under argon atmosphere. 2mmol of Se in 2 ml of TOP was injected, and the reaction was maintained at 250 °C for 5 min. Finally, the reaction mixture was cooled to room temperature, and an extraction procedure was used to purify the nanocrystals from side products and unreacted precursors. The obtained CdSe core (558 nm) was dispersed in hexane.

1.5.2 Synthesis of CdSe/ZnS core-shell QDs

The core-shell QDs synthesized by typical tri-n-octylphosphine-assisted successive ionic layer adsorption and reaction (TOP-SILAR) were described in our precious research.[6] The same process as 1.2.4 was used and the emission peak with 564 nm was obtained.

1.6 Fabrication of QDs-PS composite plate

The physical mixing method was used to form a polymer plate with green-emitting TSQDs as reference.[12,13] Typically, polystyrene (PS) polymer particles (1g) was dissolved in chloroform (3 ml), and then mixed with the prepared QDs solution (1 ml). The QDs-PS composite plate was obtained after natural drying in the mold for more than 20 h under argon atmosphere, and then compression molding by a vulcanizing machine (4MPa) at 100 °C for about 10 min.

1.7 Assembling of TSQDs-LED

The blue In-GaN/GaN LEDs with the peak emission at 455 nm were used. The QDs-PS composite plate was first mixed with silica gels (silicone 6550 gel A/gel B = 1:1) and then pasted onto the SMD-type LED. The LED chip was allowed to cure at 100 °C (30 min) so as to obtain the TSQDs based LED.[13] The EL spectra were tested in an integrated sphere and a spectrograph system.

1.8 Thermal stability test of QDs

The QDs-toluene solution was dropped on quartz plate to form a uniform film. The sample was packaged by another quartz plate using the UV curing adhesive. The QDs thermal stability sample was test under heating at different time, and then the sample is placed at room temperature for the determination of quantum yield.

1.9 Characterization

The TEM pictures were taken using a Tecnai G2 F20 S-TWIN microscope (200 KV). The UV/vis absorption spectrum of each sample was measured using a TU-1901 double-beam UV/vis spectrophotometer (Beijing Purkine General Instrument Co. Ltd., China), and the PL spectra were recorded on a fluoroSENS spectrophotometer (Gilden Photonics). The absolute PLQYs of the QDs solutions were measured using an Ocean Optics FOIS-1 integrating sphere coupled with a QE65 Pro spectrometer.[14,15] All optical measurements were performed at room temperature under ambient conditions. The electroluminescence (EL) spectra, Commission Internationale de l'Eclairage (CIE) chromaticity coordinates of the as-fabricated TSQDs-LED were measured using an ATA-500 Spectral Radiation Analyzer (EVERFINE Corporation) with an integrating sphere at room temperature.

References

1 Shen, H. *et al.* Highly Efficient Blue–Green Quantum Dot Light-Emitting Diodes Using Stable Low-Cadmium Quaternary-Alloy ZnCdSSe/ZnS Core/Shell Nanocrystals. *ACS Applied Materials & Interfaces* **5**, 4260-4265, (2013).

2 Qu, L. & Peng, X. Control of Photoluminescence Properties of CdSe Nanocrystals in Growth. *J. Am. Chem. Soc.* **124**, 2049-2055, (2002).

3 Hao, J.-j., Zhou, J. & Zhang, C.-y. A tri-n-octylphosphine-assisted successive ionic layer adsorption and reaction method to synthesize multilayered core-shell CdSe-ZnS quantum dots with extremely high quantum yield. *Chem. Commun.* **49**, 6346-6348, (2013).

4 Lei, Y. *et al.* 4 - 4: High Stability Green Luminescent Microspheres based on Quantum Dot. *SID Symposium Digest of Technical Papers* **49**, 32-35, (2018).

5 Li, J. J. *et al.* Large-Scale Synthesis of Nearly Monodisperse CdSe/CdS Core/Shell Nanocrystals Using Air-Stable Reagents via Successive Ion Layer Adsorption and Reaction. *J. Am. Chem. Soc.* **125**, 12567-12575, (2003).

6 Ren, C., Hao, J., Chen, H., Wang, K. & Wu, D. Prepare core–multishell CdSe/ZnS nanocrystals with pure color and controlled emission by tri-n-octylphosphine-assisted method. *Appl. Surf. Sci.* **353**, 480-488, (2015).

7 Yu, W. W., Qu, L., Guo, W. & Peng, X. Experimental Determination of the Extinction Coefficient of CdTe, CdSe, and CdS Nanocrystals. *Chem. Mater.* **15**, 2854-2860, (2003).

8 Reiss, P., Protière, M. & Li, L. Core/Shell Semiconductor Nanocrystals. *Small* **5**, 154-168, (2009).

9 Xiao, X. *et al.* Improving the modulation bandwidth of LED by CdSe/ZnS quantum dots for visible light communication. *Opt. Express* **24**, 21577-21586, (2016).

10 Jun, S. & Jang, E. Bright and Stable Alloy Core/Multishell Quantum Dots. *Angew. Chem.* **125**, 707-710, (2013).

11 Gao, D. *et al.* Highly Bright and Compact Alloyed Quantum Rods with Near Infrared Emitting: a Potential Multifunctional Nanoplatform for Multimodal Imaging In Vivo. *Adv. Funct. Mater.* **24**, 3897-3905, (2014).

12 Chen, W. *et al.* High efficiency and color rendering quantum dots white light emitting diodes

optimized by luminescent microspheres incorporating. *Nanophotonics* **5**, 565-572, (2016).
13 Chen, W. *et al.* Highly Efficient and Stable Luminescence from Microbeans Integrated with Cd-Free Quantum Dots for White-Light-Emitting Diodes. *Particle & Particle Systems Characterization* **32**, 922-927, (2015).
14 Mastronardi, M. L. *et al.* Size-Dependent Absolute Quantum Yields for Size-Separated Colloidally-Stable Silicon Nanocrystals. *Nano Lett.* **12**, 337-342, (2012).
15 Dai, X. *et al.* Solution-processed, high-performance light-emitting diodes based on quantum dots. *Nature* **515**, 96-99, (2014).
